# Environment-Friendly Poly(2-ethyl-2-oxazoline) as an Innovative Consolidant for Ancient Wall Paintings

**DOI:** 10.3390/nano8090649

**Published:** 2018-08-23

**Authors:** Yuanyuan Zhang, Xuanhua Li, Bingqing Wei

**Affiliations:** 1 State Key Laboratory of Solidification Processing, Center for Nano Energy Materials, School of Materials Science and Engineering, Northwestern Polytechnical University and Shaanxi Joint Lab of Graphene (NPU), Xi’an 710072, China; evazh165@163.com; 2 Department of Mechanical Engineering, University of Delaware, Newark, DE 19716, USA

**Keywords:** organic consolidation material, poly(2-ethyl-2-oxazoline), eco-friendly, Paraloid B72, wall paintings conservation

## Abstract

The research of innovative materials on the conservation of ancient wall paintings has given rise to increased attention in recent years. One of the most used synthetic organic consolidation material for the wall paintings is the commercial acrylic resin Paraloid B72 (PB 72), which encounters problems of the use of toxic solvents, low water vapor transmission, and poor penetration. Here, the non-toxic, environment-friendly product poly(2-ethyl-2-oxazoline) (PEOX) has been demonstrated as a great potential consolidant for wall paintings to solve these issues. First of all, thanks to the better penetration ability, the simulating plaster sample treated with PEOX shows greater enhanced surface hardness than PB 72. The single-lap joint shear strength test and the scotch tape test revealed the good adhesion of PEOX on inorganic surfaces and effective pigment consolidation. At the same time, the PEOX-treated sample presents less surface gloss. The hydrophilic nature of PEOX merits itself with superior water vapor permeability compared with PB 72. These advantages enable PEOX to be a progressive choice to replace the use of PB 72 in the controlled indoor working environment.

## 1. Introduction

Wall paintings are a type of art painted on the walls and/or ceilings of caves, buildings, or tomb chambers, which can be dated back to more than 64,000 years ago [1]. In recent decades, the conservation and diagnosis of cultural heritage have attracted more and more attention from the fields of materials science [2,3,4]. For example, the nanoscale materials are adopted in the cleaning and consolidation of ancient monuments with an increasing trend [5,6,7]. Compared with other types of historic objects, the conservation of wall paintings is a crucial challenge since the complicated structure of wall paintings consists of both inorganic materials and organic components (e.g., pigments, glue, supporting layers, etc.). Therefore, several factors should be taken into consideration for ideal conservation materials under different circumstances.

**Figure 1:**
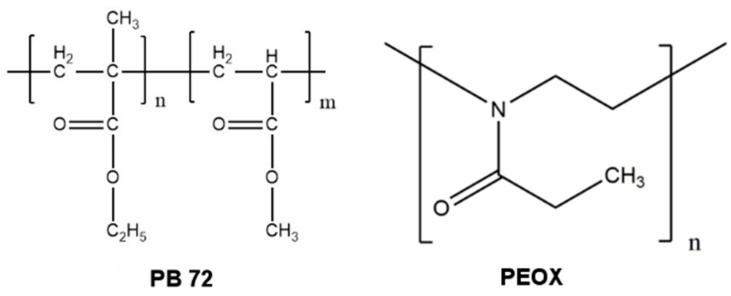
The molecular structure of Paraloid B72 (PB 72) and poly(2-ethyl-2-oxazoline) (PEOX), respectively.

Currently, the organic synthetic materials that are mostly adopted for the consolidation of cultural heritage are acrylic polymers because of their transparent film-forming ability and good adhesion. Paraloid B72 (PB 72), which is a commercial product with chemical composition of poly(ethyl methacrylate-methyl acrylate) (70/30) (Figure 1), was widely introduced to the consolidation of cultural heritage in the 1970s due to its outstanding weathering stability compared with other acrylic polymers [8]. A large number of case studies and restoration reports have proven PB 72 as a successful consolidant for wall paintings after long-time practical trials [9,10,11]. However, some drawbacks of PB 72 should be of concern as well. First of all, acetone, toluene, or xylene are usually used as a solvent to dissolve PB 72 at various concentrations. PB 72 is hardly soluble in common alcohols including ethanol and isopropyl alcohol. In the indoor environment without good ventilation, these solvents are quite harmful to the health of the operators. Moreover, wall paintings which are treated by PB 72 often present a shiny film on the surface layer. Several studies have reported that this film hinders the interaction between wall painting and the environment [12]. 

Poly(2-ethyl-2-oxazoline), which is often abbreviated as PEOX or PEOz, is a tertiary amide polymer polymerized by cationic ring-opening polymerization of 2-ethyl-2-oxazoline (Figure 1). Commercial products of PEOX, namely Aquazol, has a wide variety of solubility in common organic solvents including alcohols and ketones. In addition, PEOX has been reported with good adhesion on different surfaces including aluminum foil, nylon, poly(methyl methacrylate) (PMMA), and polyvinyl alcohol (PVA) [13]. Furthermore, due to its low cytotoxicity and “living polymerization” characteristic, PEOX has been applied in electronic devices [14,15] biomedical applications including drug delivery [16], anti-fouling coatings individually or as a monomer of block copolymers [17,18,19]. PEOX can also be adopted as a coating material because of its good light stability and re-solubility [20,21]. However, up to now, the environment-friendly material PEOX has seldom been reported and evaluated as a consolidant for wall paintings.

In the present work, we reported the use of PEOX as a consolidant for wall paintings and evaluated the applicability of PEOX extensively. We have investigated the mechanical property and the consolidation effect of PEOX on both the film and the simulating wall painting samples. The data implied that the consolidation efficacy of PEOX is higher than PB 72. The mechanisms of the promising consolidation efficacy are discussed from the aspects of mechanical strength, penetration depth, and adhesion strength. In the end, the water vapor permeability and color change in the laboratory are also monitored. The use of PEOX as consolidant avoids harsh solvents, and the convincing consolidation effects suggest that PEOX is an advantageous alternative resin of PB 72 in a dry environment.

## 2. Materials and Methods

### 2.1. Materials

Poly(2-ethyl-2-oxazoline) was purchased from Alfa Aesar (Shanghai, China) Chemicals Co. Ltd. Mw = 200,000. Paraloid^®^ B72 was provided by Shaanxi History Museum. The two resins were used as received. The solvent acetone and isopropyl alcohol were obtained from Sinopharm Chemical Reagent Co., Ltd. (Shanghai, China). PEOX was dissolved in isopropyl alcohol, and PB 72 was dissolved in acetone with varying concentration when applied on samples.

### 2.2. Simulating Sample Preparation

**Figure 2:**
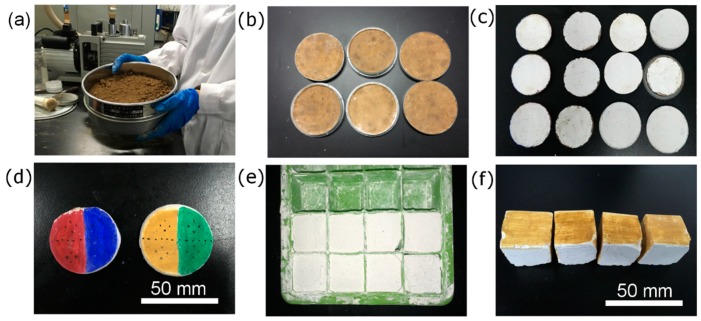
Preparation procedures of the simulating samples: (**a**–**d**) Preparing the rounded samples, (**e**,**f**) Preparing the cubic samples.

The rounded samples consisted of two layers were molded in polystyrene (PS) Petri dishes with a diameter of 60 mm by the following procedures to imitate the ancient wall painting structure (Figure 2a–d). The ancient wall painting mainly consists of ground layer, plaster layer and pictorial layer. First, the earth for the ground layer was sieved through the 30-mesh sieve and was mixed with a certain amount of water to make fine mud layer. The plaster layer was mainly wet slaked lime (Ca(OH)_2_) with a trace amount of silicate impurities. A small amount of short hemp fibers was added into the wet slaked lime in order to ease the shrinkage of the plaster layer during drying. Finally, the mud was mold in the Petri dish followed by the fine plaster layer. The thickness of the ground layer and the plaster layer were approximately 5 mm and 3 mm, respectively. Simulating wall painting samples for the scotch tape test were made by molding wet slaked lime in a silicone mold having a size of 25 × 25 × 25 mm^3^ (Figure 2e,f). All the samples were then left in room ambient for a month before further tests. The wet slaked lime of the sample reacted with CO_2_ and became plaster CaCO_3_ (carbonation). The pigments are painted after the carbonation of the samples and the pigments used here are cinnabar (red), ultramarine (blue), yellow ochre (yellow), and malachite (green) (Jiang Sixu Tang Company, Suzhou, China).

### 2.3. Characterization

**Figure 3:**
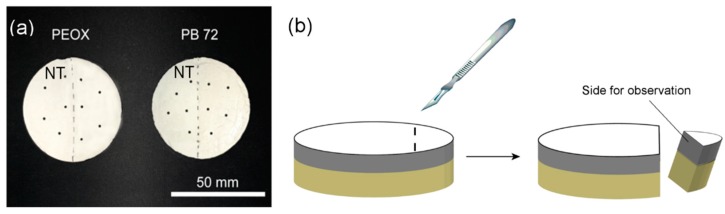
(**a**) The samples for the surface indentation test, black dots refer to the test positions and “NT” stands for the not treated area; (**b**) Preparation of the cross-sections.

The hardness of the simulating samples was measured using the Equotip^®^ 3 (Proceq Company, Schwerzenbach, Switzerland) portable hardness tester on the rounded samples, and the tests were executed with the C-type impact device with an impact energy of 3 Nmm. As shown in Figure 3a, the left parts of the rounded samples were not treated (NT). The right parts were brushed firstly by the consolidant with a concentration of 2.5% for three times and subsequently brushed by the consolidant with a concentration of 5% for one time in order to get maximum penetration and sufficient consolidation. Five tests were made on the treated part of the samples for each consolidant. The hardness was expressed in Leeb hardness (HLC).

For the tensile strength test, the films were made by pouring the solution into a Polytetrafluoroethylene (PTFE) mold, which has a volume of 50 × 50 × 5 mm^3^. After drying at 50 °C for 48 h, the films were peeled off carefully by a scalpel. The film samples were then cut into 50 × 5 mm^2^, and the gauge length was 10 mm during the test. The thickness of the films of PEOX and PB 72 was about 90 µm and 140 µm, respectively. The tensile strength of PEOX and PB 72 was performed on Instron 5942 universal testing machine (Instron Engineering Corporation, Norwood, MA, USA) equipped with a 500 N load cell and with the test speed at 5 mm/min. Each material was tested for ten replicas, from which the mean values and standard deviations were calculated. With the consideration of the high hygroscopicity of PEOX reported by Arslanoglu and Muros [22,23], the mechanical tests were performed in lab ambient of 23 °C and RH 50–60%. Film hardness tests were performed by using QHQ-A pencil hardness tester (Aipu Insruments Co. Ltd., Quzhou, China) according to ISO 15184:2012 at a temperature of 17 °C and RH 42%. Briefly, in the pencil hardness test, the pencil with a known hardness grade (from 6B to 6H) scratches on the coating film, and the hardness of the film is evaluated according to the pencil which can make damage trace on the film. The softest grade of the pencil represents the hardness of the film.

The morphology of the samples was studied by scan electron microscopy (SEM) (FEI Nova NanoSEM 450, FEI Company, Hillsboro, OR, USA). For the penetration depth analysis of PEOX and PB 72 on plaster, the cross-sections were taken from the simulating samples after the hardness test (Figure 3b). The stratigraphic distribution of the consolidants was mapped by using Thermo Nicolet iN10 Fourier Transform Infrared spectroscopy (FTIR) imaging microscope (Thermo Fisher Scientific, Waltham, MA, USA). The spectra were recorded in the reflection mode with a spectral resolution of 8 cm^−1^ in the range between 4000–675 cm^−1^ with an aperture 50 × 50 µm. Thirty-two scans were taken for each point of the measuring area on the samples. The surface gloss of the simulating samples was recorded using 3nh^®^ HG268 gloss meter (3nh Company, Shenzhen, China) at 60 °.

To investigate the consolidation effectiveness of PEOX and PB 72 for pigments, the scotch tape tests were performed on the cubic plaster samples. Five percent PEOX and PB 72 were brushed on the painted surfaces, respectively. A strip of pressure-sensitive tape with a width of 28 mm, was put on the treated surface. About 90 s later, the tape strip was pulled off at an angle of 90 ° with a speed of about 10 mm/s. The amount of pigment loss was weighed to the precision of 0.1 mg. The tests of each material were repeated on five painted surfaces. The aluminum specimens for the single-lap joint shear strength were 50 × 12 mm in size, and the bonded area is 12 × 6 mm. Before bonding, the aluminum specimens were washed with acetone.

The water vapor transmission rate of PEOX and PB 72 treated samples were evaluated according to ASTM-D1653. The home-made experiment equipment set was prepared as follows: the flask was filled with about 75 mL distilled water, and the rounded samples were ground into a diameter of 40 mm to fit the size of the flask. Then the samples were put on the flasks with the treated side down. The gap between the sample and flask was sealed by parafilm and microcrystalline wax. The plaster samples were previously brushed with 5% PEOX and 5% PB 72, respectively, and each material had three replicas. The untreated samples were chosen as the control. The sets were kept in the test chamber (T = 25 °C and RH = 50 ± 2%) and weighed every 24 h.

X-rite vs450 benchtop sphere spectrophotometer (X-Rite Inc., Grand Rapids, MI, USA) was used to monitor the colorimetric difference brought by PEOX on painted samples. The color scale was mathematically expressed in the three-dimensional CIE L*a*b* colorimetric space (CIELAB) with standard illuminant D65 and 10 ° observer degree. The color difference of each sample was calculated by the equation below.

(1) $$\Delta E=\sqrt{\Delta {L*}^{2}+\Delta {a*}^{2}+\Delta {b*}^{2}}$$

where ΔL*, Δa*, and Δb* are defined as the coordinate change of lightness, green-red, and blue-yellow in the colorimetric space, respectively.

## 3. Results and Discussion

A brief comparison between PEOX and PB 72 is demonstrated in Table 1 in addition to the properties to be tested in this study related to the consolidation of wall paintings.

### 3.1. The Mechanical Strength Tests

**Figure 4:**
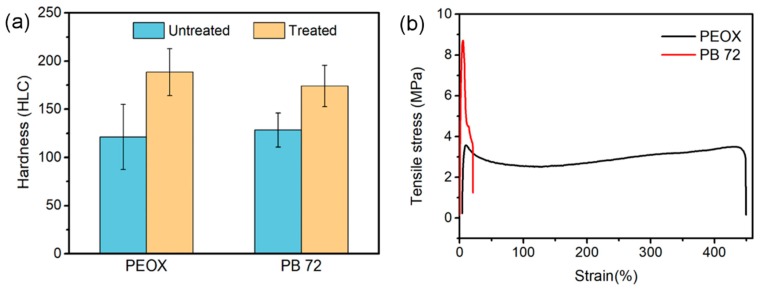
(**a**) The hardness values of the untreated area and treated area; (**b**) Tensile stress-strain curves of PEOX and PB 72 (curves that are mostly close to the average values are selected to represent each consolidant).

The consolidation strength was measured by the hardness test on the simulating samples. The measurements were done on five different positions on the simulating sample. As shown in Figure 4a, after treated with PEOX, the average hardness of the sample is raised dramatically from 121.4 HLC to 188.6 HLC (55.3%). The average surface hardness of PB 72 treated sample is also improved from 128.4 HLC to 174.2 HLC (35.7%) as expected. These results imply that PEOX is a more effective consolidation material.

The consolidation strength is influenced by the mechanical strength of the consolidant itself as well as the penetration depth of the consolidant. To confirm the origin of the effective consolidation in PEOX, we first study the mechanical strength of the consolidant. As shown in Figure 4b and Table 2, the tensile stress at yield of PEOX is lower than PB 72, and the strain at yield of PEOX is slightly higher than PB 72. Moreover, the strain at break of PEOX is 501.67%, which is much higher than PB 72 (27.45%). Furthermore, the pencil hardness of the PEOX film is assigned to HB, while that of PB 72 is H (Table 2). (HB and H refer to the hardness grade of the pencil’s core, HB is softer than H.) These results indicate that PEOX is softer and more ductile, while PB 72 is more rigid and brittle. From the polymer structure, the main chain –N–C– of PEOX is more flexible than the –C–C– chain of PB 72. Moreover, in the room ambient (RH ≥ 50%), the water within PEOX acts as the plasticizer and makes PEOX more plastic [24]. Thus, the effective consolidation in PEOX is not attributed to the mechanical strength of the PEOX.

**Figure 5:**
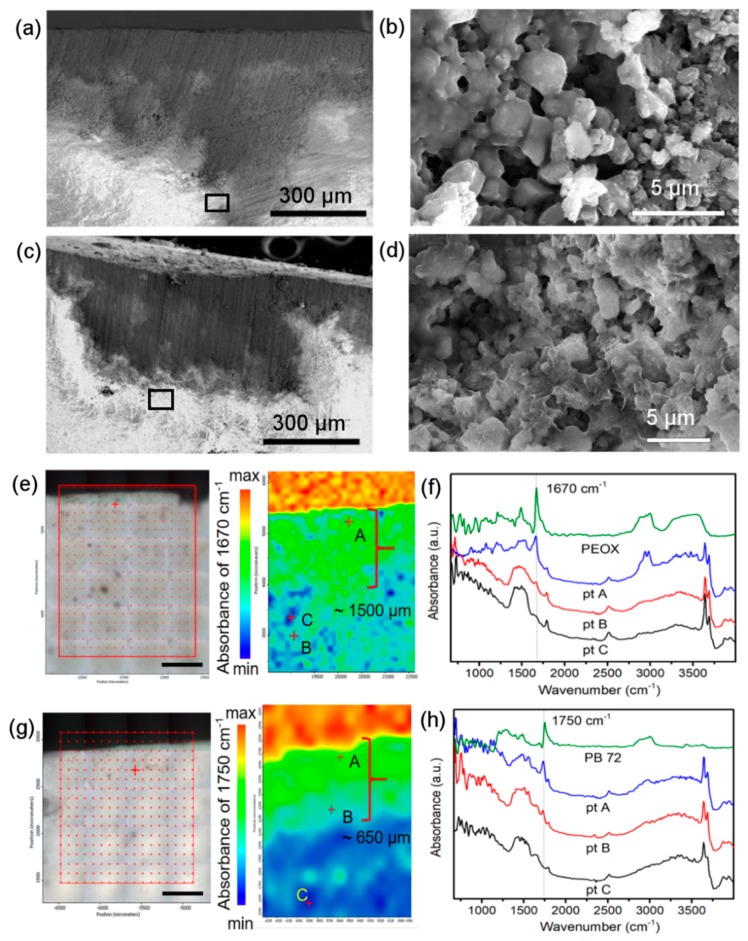
(**a**,**c**) The cross-sectional scan electron microscopy (SEM) images of the samples treated with PEOX and PB 72, respectively; (**b**,**d**) Magnification of the black frame in (**a**,**c**), respectively; (**e**) Optical micrograph of Transform Infrared spectroscopy (FTIR) mapping analysis area on PEOX treated sample and corresponding false-color map of 1670 cm^−1^. The alphabets refer to the analysis points (scale bar: 500 µm); (**f**) FTIR spectra of bulk PEOX and different analysis points in (**c**); (**g**) Optical micrograph of FTIR mapping analysis area on PB 72 treated sample and the corresponding false-color map of 1750 cm^−1^. The alphabets refer to the analysis points (scale bar: 500 µm); (**h**) FTIR spectra of bulk PB 72 and different analysis points in (**g**). (pt = point).

To investigate the reasons that cause the different consolidation strengths, we studied the penetration depths of PEOX and PB 72. Figure 5a,c present the morphology of the cross-section of the PEOX and PB 72 treated samples respectively. The graphs distinctly show that the upper dark part of the samples is penetrated with the organic consolidants. The magnification graphs in Figure 5b,d illustrate the deepest boundary of the penetration of PEOX and PB 72, respectively. In addition, it is clearly shown that the particles of the plaster are connected by the consolidant films. The infrared microscopy imaging system was adopted here to chemically investigate the penetration depth of PEOX and PB 72. In the case of PEOX, the peak at 1670 cm^−1^, which is assigned to the C=O stretching of the amide I band, was taken as the characteristic peak for the mapping analysis of PEOX [25]. Combing Figure 5e and the spectra in Figure 5f, it is clear that the absorbance intensity of C=O stretching is strong along the depth of nearly 1500 µm from the surface of the sample, and the weak absorbance can also be detected in some deeper area (Figure 5e point B). The result indicates that PEOX has a good penetrating ability to a deep position. The chemical mapping of PB 72 was recorded in Figure 5g. Similarly, the absorbance peak at 1750 cm^−1^ was selected as the characteristic peak, which is the C=O stretching band of the carbonyl group of PB 72 [26]. As shown in Figure 5g,h, the spectrum of point A, which is close to the surface of the sample, presents the strongest absorbance at 1750 cm^−1^. The absorbance of this peak at point B decreases a little, whereas the point C shows no absorbance of PB 72. It can be concluded that PB 72 is distributed in the depth of about 650 µm. It is worth clarifying that the red areas on the top of the false color images in Figure 5e,g refer to the sample stage but not the sample. Because during the measurements, the absorbance baseline of the spectra recorded at the sample stage is higher than the sample area. Thus, the intensity values of these areas seem bigger than the sample area. These mapping results exhibit that PEOX in isopropyl alcohol has a better penetration ability than PB 72 in acetone. To ensure an effective consolidation result, the consolidant should penetrate into the wall painting to a sufficient depth rather than just remaining on the surface layer, especially for the severely deteriorated wall paintings [27]. Therefore, it can be inferred that the greater penetration of PEOX contributes to the better consolidation strength compared with PB 72 even though the mechanical strength of PEOX itself is softer and more plastic.

Furthermore, the possible reasons for the superior penetration of PEOX are discussed here. Besides gravity, capillary force is the main power that drives the liquid into the porous substrate. Meanwhile, considering the similar porosity of the samples, the factors that fundamentally force the capillary penetration include wettability and surface tension. PEOX has a more polar structure compared with PB 72, and the polarity of the solvent isopropyl alcohol is slightly higher than acetone as well. The stronger interaction between PEOX and the sample makes PEOX spread better than PB 72 on the surface of calcite, which brings polar groups. On the other hand, the surface tension of isopropyl alcohol is slightly lower than acetone, which promotes the capillary penetration in the high surface energy calcite [28]. Another possible reason that causes the penetration difference could be that acetone has a quicker evaporation rate than isopropyl alcohol. Before PB 72 penetrating into a deeper position, acetone has already evaporated and makes PB 72 to stay in a relatively shallower depth. The penetration might be improved if PB 72 dissolved in a less volatile solvent, e.g., toluene or a mixture of acetone and ethanol [29], but the choice for the non-harsh solvent is very limited, unfortunately. In contrast, the broad solvent choice of PEOX takes advantage in controlling the penetration depth.

### 3.2. Gloss Measurement of the Consolidated Samples

The glossy film formed on the surface of wall painting after the consolidation treatment is a common drawback of organic consolidants and thereby alters the appearance of wall paintings. The surface gloss of the bulk simulating samples brushed by PEOX and PB 72 was studied. The original gloss of the plaster layer is 2.46 ± 0.11 gloss units (GU). After being brushed with PEOX, the gloss raises slightly to 4.90 ± 0.81 GU. By comparison, the surface gloss significantly increases to 10.18 ± 0.93 GU after brushed with PB 72. Obviously, the poor penetration results in the increment of surface gloss. The superior penetration ability of PEOX endorses not only sufficient consolidation of the porous plaster layer of wall paintings but also offers less surface gloss on the pictorial layer.

### 3.3. The Tests of Pigment Consolidation

**Figure 6:**
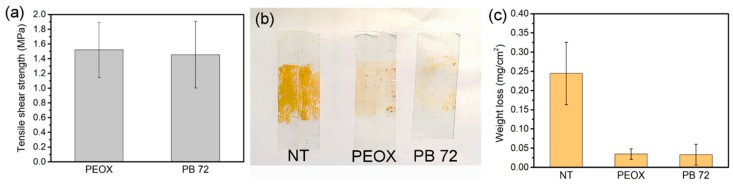
(**a**) Single-lap joint shear strength of PEOX and PB 72; (**b**) Consolidation efficiency test by tape test on painted samples. “NT” stands for the not treated sample; (**c**) Weight loss of pigment on the untreated sample and the sample treated with PEOX and PB 72 respectively by the Scotch tape test.

A “bridge” between the consolidant and the particles of wall painting is needed in the consolidation treatment to connect the deteriorated coarse particles. Thus, the consolidation materials require adequate adhesion on the surface of the wall painting. The single-lap shear strength of PEOX and PB 72 were tested on aluminum specimens in order to evaluate the adhesion strength (Figure 6a). The lap-shear strength of PEOX is 1.52 ± 0.48 MPa, and PB 72 is 1.45 ± 0.39 MPa, respectively. The results have revealed the good adhesion of PEOX on metal and shown that the adhesion of PEOX is comparable to that of PB 72. Moreover, the consolidation of pigment on plaster was evaluated by the Scotch tape test after the simulating samples were painted with yellow ochre (Figure 6b,c). The untreated sample was selected as the control in this test. In the case of the untreated sample, the pigment grains removed by the adhesive tape was 0.24 ± 0.080 mg/cm^2^. After being consolidated with PEOX, the pigments were adhered and fixed on the surface by the PEOX film, and the loss of pigment decreased dramatically to 0.035 ± 0.014 mg/cm^2^. The result of PB 72 (0.033 ± 0.027 mg/cm^2^) was more or less equal to that of PEOX. From the chemical structure point of view (Figure 1), we can see that the tertiary amide main chain and the carbonyl group in PEOX are hydrogen bond acceptors. Therefore it can be inferred that the good adhesion ability of PEOX comes from the hydrogen bonds between PEOX and the adhered surface.

### 3.4. Water Vapor Permeability

**Figure 7:**
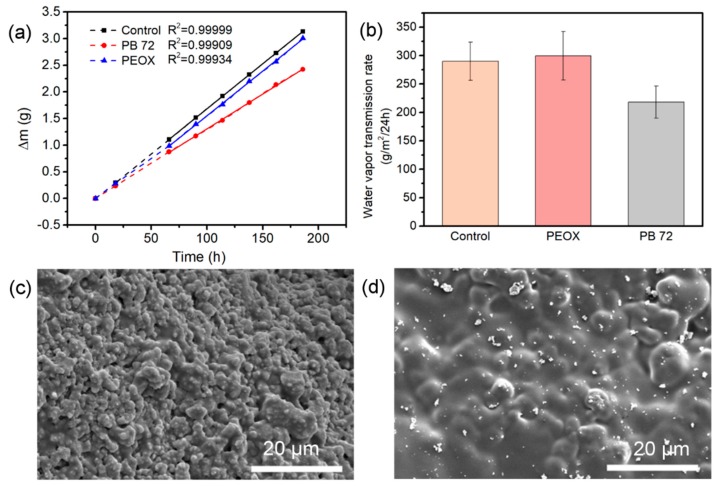
(**a**) The curves of weight change of the set in each 24 h with test time; (**b**) The water vapor transmission rate of the untreated samples, PEOX treated samples, and PB 72 treated samples for 120 h; (**c**,**d**) SEM images of the morphology of the surface of PEOX treated sample and the PB 72 treated sample after the test, respectively.

In the most cases, the water within the wall paintings exchanges with the outer environment in the form of vapor through the porous structure. Sometimes, the synthetic polymeric conservation material deposited on the surface of wall paintings will hinder the water vapor exchange, which leads to the gathering of soluble salts and eventually causes further deterioration on wall paintings [30]. To investigate how the water-soluble resin PEOX affects the water vapor transport, the test of the water vapor transmission rate was done, and the result was compared with PB 72. For each tested material, plot the weight change (Δm) against time (h). When a straight line fits the plot of at least four points of the certain time interval, the test system reaches a steady state (Figure 7a). Then the water vapor transmission rates are calculated from the straight line from 66 h to 186 h using the following equation:

(2)  Water vapor transmission rate =ΔmAt

where Δm: weight change, g (from the straight line), A: test area, m^2^, and t: 24 h during which Δm occurred. 

As shown in the histogram graph (Figure 7b), the water vapor transmission rate of the PEOX treated samples are nearly equal to that of the controls, indicating that PEOX brings almost no change on the water vapor exchange behavior. By contrast, the water vapor transmission rate of the PB 72 treated samples decreases obviously. PEOX, as a hydrophilic resin, permits water vapor diffusing and passing through the film. While PB 72 is a hydrophobic resin, of which the film formed on the surface of the sample blocks the pores of the sample and reduces the water vapor transmission. The SEM graph of the PEOX treated sample after the water vapor transmission test (Figure 7c) demonstrates that the coarse grains of plaster sample are connected by the PEOX film, and the porous morphology of sample has not been modified by PEOX dramatically. On the contrary, the film of PB 72 strongly changes the surface feature of the sample and lowers the porosity of the sample (Figure 7d). To sum up, the use of PEOX will not affect the normal water vapor exchange between wall painting and the environment significantly.

### 3.5. Colorimetric Difference Monitoring

The aesthetic appearance of cultural heritage, especially ancient objects of fine arts, should not be altered by the conservation treatment. Therefore, the L*, a*, and b* coordinate values of four different colors on simulating samples were recorded before the treatment and after the samples were brushed with 5% PEOX. After being stored near the window of the laboratory for six months, these samples were investigated again to monitor the color change on the PEOX treated pigments (Table 3). The color difference value ∆E of each color was calculated from the original color and the color after the treatments. The overall ∆E of the four colors immediately after the treatment is less than 3, which cannot be recognized by naked eyes [31]. The L* value of all the four color decreases slightly due to the saturation effect of PEOX. Moreover, probably because PEOX is naturally pale yellow and hence makes the b* value of these colors increase the most among the three coordinates. In addition, the natural aging brings almost no change in colors after six months. In summary, the consolidation of wall paintings by PEOX is acceptable in terms of the aesthetic aspect.

## 4. Conclusions

This research introduced the environment-friendly PEOX as an innovative consolidant for the ancient wall paintings and systematically studied the properties of PEOX on the films and simulating samples. Above all, the surface hardness test showed that the enhancement of the average hardness value of the PEOX consolidated simulating sample is bigger than the common consolidant PB 72 under the same consolidation procedures. The good penetrating ability of PEOX is the major merit that results in this more effective consolidation rather than the mechanical strength of the consolidant itself. The deeper penetration depth of PEOX is mainly due to better wettability and the use of the solvent with lower surface tension and a slower evaporation rate. The desirable penetration depth enables PEOX suitable for the consolidation of wall paintings, especially for the plaster ground layer and brings less gloss on the surface of wall painting. Moreover, it was proved that PEOX has good adhesion on aluminum surface by single-lap joint shear strength test, and the tape test results also showed adequate consolidation effect of pigments on the plaster layer. Additionally, due to its hydrophilic property, the water vapor transmission in PEOX-treated samples was revealed to be more outstanding than PB 72 because PEOX will not change the porosity of wall painting dramatically, as PB 72 does. The six-month CIELAB colorimetric difference monitoring suggested that PEOX does not alter the aesthetic appearance of the pictorial layer. Due to the several advantages, PEOX is recommended as a promising consolidant during the restoration and conservation of wall paintings in the humidity controlled environment, and with no doubt more practical experiments on ancient wall painting fragments should be implemented in the future.

## Figures and Tables

**Table 1:** Basic properties of poly(2-ethyl-2-oxazoline) (PEOX) and Paraloid B72 (PB 72) reported in the literature.

Properties	PEOX	PB 72 [3]
Refractive index	1.52 [6]	1.48
Color	Pale yellow	Colorless
Glass transition temperature	55 °C on quenched sample, 69–71 °C on unquenched sample [6]	40 °C
Softening range	110–120 °C [6]	Approx. 70 °C
pH	6.4 ± 0.1 [11]	

**Table 2:** The tensile strength of the film of PEOX and PB 72.

Sample	Tensile Stress at Yield [MPa]	Strain at Yield [%]	Tensile Stress at Break [MPa]	Strain at Break [%]	Pencil Hardness
PEOX	3.76 ± 0.47	8.07 ± 1.17	3.14 ± 0.18	501.67 ± 68.72	HB
PB 72	7.60 ± 1.01	5.90 ± 0.77	3.29 ± 0.23	27.45 ± 6.67	H

**Table 3:** Colorimetric change monitoring on different colors after treated by PEOX immediately and after six months.

Color	Test Time	L*	a*	b*	∆E
Red	Before treatment	37.45	47.68	20.82	-
	Immediately after treatment	37.03	47.59	22.45	1.69
	6 months after treatment ^1^	36.64	47.53	22.56	1.93
Blue	Before treatment	41.49	21.31	−61.03	-
	Immediately after treatment	41.35	22.28	−60.22	1.27
	6 months after treatment	41.43	22.41	−59.78	1.67
Yellow	Before treatment	63.53	21.16	52.56	-
	Immediately after treatment	62.89	20.63	54.24	1.87
	6 months after treatment	62.59	20.05	54.49	2.42
Green	Before treatment	59.4	−50.17	14.56	-
	Immediately after treatment	57.96	−50.22	15.06	1.53
	6 months after treatment	57.79	−50.59	15.10	1.75

^1^ The ΔE after aging was calculated by comparing with the samples before treated.
